# Devising a Coaching Method for a Smartphone-Based Slit-Lamp Microscope and Its Learning Effects: A Pilot Study

**DOI:** 10.3390/jcm15051928

**Published:** 2026-03-03

**Authors:** Hokuto Ubukata, Haruo Toda, Hiroki Nishimura, Shintaro Nakayama, Mai Nishio, Takahiro Mizukami, Kosei Tomita, Eisuke Shimizu

**Affiliations:** 1 Department of Orthoptics and Visual Sciences, Faculty of Medical Technology, Niigata University of Health and Welfare, Niigata 950-3198, Japan; toda@nuhw.ac.jp; 2 Major in Health and Welfare, Graduate School of Health and Welfare, Niigata University of Health and Welfare, Niigata 950-3198, Japan; 3 Department of Ophthalmology, Keio University School of Medicine, Tokyo 160-8582, Japan; hiroki@ouiinc.jp (H.N.); p.shintaro@ouiinc.jp (S.N.); ophthalmolog1st.acek39@keio.jp (E.S.); 4 Yokohama Keiai Eye Clinic, Kanagawa 240-0065, Japan; 5 OUI Inc., Tokyo 107-0062, Japan; 6 Aoba Eye Clinic Takenotsuka, Tokyo 121-0801, Japan; mainmain1210@gmail.com; 7 Fuchu Eye Center, Fuchu Hospital, Osaka 594-0076, Japan; t_mizukami@seichokai.or.jp; 8 Department of Ophthalmology, Kawasaki Medical School, Okayama 701-0192, Japan; kt394144@gmail.com

**Keywords:** slit-lamp microscope, anterior segment, mobile device, coaching, medical education, Smart Eye Camera

## Abstract

**Objectives**: To develop an effective learning method for using a smartphone-based slit-lamp microscope (SBSL) and to identify key points to emphasize when coaching individuals with no prior SBSL experience. **Methods**: This study included 60 orthoptic students: 40 second-year students (control group: 20, training group 1: 20) and 20 first-year students (training group 2). Subjects were instructed to record the anterior eye segment of a patient-role subject using the Smart Eye Camera. The control group was given paper instruction and was shown the demonstration of the SBSL beforehand. In addition, training groups 1 and 2 watched a tutorial video, practiced using the SBSL for 30 min, and received guidance from an expert. Four ophthalmologists evaluated the recordings based on the eyelid, conjunctiva, cornea, pupil including iris, lens, and anterior chamber depth. **Results**: ANOVAs showed significant differences among groups for all items. The control group had significantly lower scores than both training groups, while no significant differences were found between training groups 1 and 2. Principal component analysis of training groups 1 and 2 showed that the first principal component accounted for 74.36% of the variance. The second principal component accounted for 10.71%, with a wide range of loadings (anterior chamber depth of 0.7780 to conjunctiva of −0.5585), implying the existence of different favorite focusing depths within subjects. **Conclusions**: A coaching program consisting of tutorial video learning, a 30 min hands-on trial, and feedback is effective in helping individuals without an ophthalmological background acquire anterior segment imaging skills using SBSL. Comprehensive focusing across the entire anterior segment should also be emphasized.

## 1. Introduction

The slit-lamp microscope is the gold standard for evaluating anterior segment structures [1,2,3], and stationary devices are the standard [4]. However, stationary instruments are not suitable for infants and patients who have difficulty maintaining posture and fixation, such as during house calls. Such stationary instruments are also difficult to use outside of ophthalmic institutions [2].

In the past, handheld slit-lamp microscopes were used outside of ophthalmology facilities [5,6]. In time, smartphones have rapidly spread in our daily lives, and the number of mobile-cellular telephone subscriptions was estimated to be 7125 million [7] to 9005 million [8] worldwide in 2024. Accordingly, the development of a smartphone-based slit-lamp microscope (SBSL) consisting of an attachment for a slit-lamp microscope has been underway [2,9,10,11]. The operation of a smartphone is simple and easy-to-use [12], and the SBSL can be used for a wide range of applications. Additionally, it is user-friendly [13], with little trouble for both SBSL operators and patients in the modern age. In addition, SBSL-compatible smartphones are commercially available, and the dedicated attachments are inexpensive, compared to existing stationary slit-lamp microscopes, making them cost-effective [12]. In addition, even most basic smartphones are equipped with camera functionality and a light source, making it possible to record the findings of the anterior segment as they are captured [2,9,14]. Moreover, with the development of communication technology, information recorded in smartphones or dedicated applications can be stored using cloud computing [12], which facilitates transmission, reception, and bidirectional exchange [15,16]. Therefore, the SBSL is mainly used in telemedicine [2,17,18] and screening in areas with limited medical resources [10]. This also allows non-eye healthcare specialists to have access to such diagnostic equipment [2,18,19] or even non-medical professionals [2], which is increasing every year [20].

Although video learning [21], simulators [22], and smartphone-mounted learning [23] have been reported for slit-lamp microscope education, the equipment used is stationary, and, to our best knowledge, education using SBSLs has not been studied. Assuming that the number of medical and non-medical personnel other than ophthalmologists who use an SBSL will further increase in the future, it is necessary to devise a short-term coaching method for capturing high-quality anterior segment findings and to clarify the issues to be emphasized in the content.

Therefore, the first objective of this study was to devise a method of learning to use an SBSL for non-healthcare professionals without medical education specifically in ophthalmology, and to test its effectiveness. The second objective was to identify issues that should be taken care of in coaching those who have no experience using an SBSL to record appropriate anterior segment findings.

## 2. Materials and Methods

### 2.1. Subjects

The subjects were 60 orthoptic students enrolled at University A, including 40 second-year students and 20 first-year students who agreed to participate in this study. The duration of enrollment was 1 year and 1 month for the second-year students and 1 month for the first-year students. None of the subjects had any prior experience using slit-lamp microscope, including SBSL. The first-year students had not yet earned credits in basic anatomy and physiology, which are foundational subjects also taught in training programs for medical staff other than orthoptists, whereas the second-year students had already completed these courses. In addition, the second-year students had already earned credits in lecture and laboratory courses on basic visual function and ophthalmic examinations and were therefore able to interpret anterior segment findings.

The 40 second-year students were further divided into the “control group” and “training group 1” of 20 students each, and the first-year students were defined as “training group 2”. The “control group” and “training group 1” were matched based on end-of-year examination scores (written and practical) and then assigned to two groups of 20 students each using a computer-generated randomization sequence. The randomization sequence was generated in advance using a pseudo-random number generator, and allocation was performed using a 1:1 ratio.

We also recruited a subject who played the patient role (“the patient-role subject”). The patient-role subject was selected among the applicants who fully understood the purpose and risks of the study.

### 2.2. Smartphone-Based Slit-Lamp Microscope Used in This Study

The design of this study was a prospective study. In this study, we used the Smart Eye Camera (SEC) (OUI. Inc., Tokyo, Japan) [9]. The SEC consists of a 3D-printed anterior segment observation attachment along with a smartphone, on which the attachment can be mounted to perform slit-lamp microscopy. The SEC leverages the light source and the camera function of the smartphone and takes slit-lamp microscope videos. The captured videos are stored in a dedicated application installed on the smartphone. The data in the application is shared with authorized users via cloud computing. Only those who are authorized to view the videos can view and comment on the shared data, as long as they have access to an Internet environment, allowing interactive communication with the operator.

### 2.3. Data Acquisition and Pre- and Post-Acquisition Procedures

**Figure 1:**
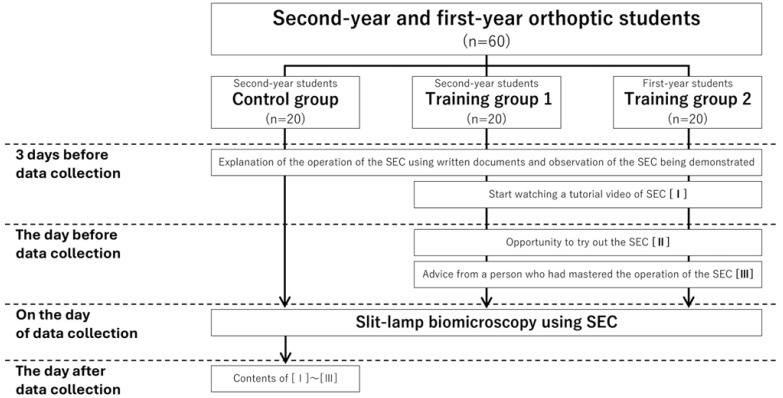
Flowchart of data acquisition for each group.

**Figure 2:**
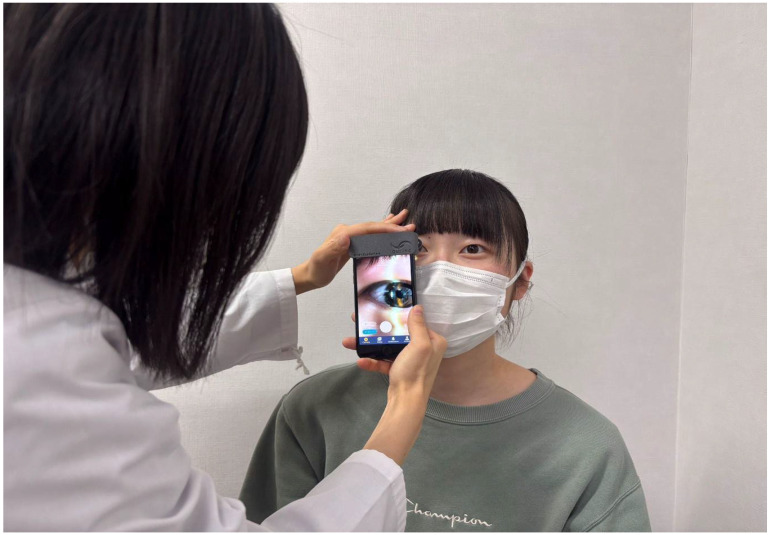
Taking video of the anterior segment using the SEC. The upper side of the application screen displays the shooting time, and the lower side has buttons to start and end shooting.

The flow of data acquisition for each group is shown in Figure 1. Three days before data acquisition, subjects in all groups were instructed to record anterior segments using the SBSL’s slit light on the patient-role subject. In addition, they received written instructions on the operation of the SEC and observed an experienced user operate the SEC. In addition, training groups 1 and 2 began watching a 6 min tutorial video (https://m.youtube.com/watch?v=AFFWkdTV-Dw&feature=youtu.be (accessed on 10 January 2026)) (Table 1), available only to SEC users via YouTube, starting from three days prior to data acquisition (Figure 1 [I]). The tutorial video was distributed with a viewing URL and QR code so that it could be viewed repeatedly. In addition, training groups 1 and 2 were given an opportunity to try out the SBSL for 30 min on the day before the examination (Figure 1 [II]). During the trial period, subjects practiced in pairs with each other and received advice and feedback from the experienced SEC user (HU). All groups were instructed to take pictures with the slit light at the center of the eyeball. Then, they were instructed to make two to three sweeps from the inner canthus to the lateral canthus for each eye. They had to repeat these maneuvers until they thought that they had obtained satisfactory videos (Figure 2).

The data acquisition was performed on both eyes of a 22-year-old female without organic ocular diseases (the patient-role subject). The following conditions were imposed on the subjects: the maximum acquisition time was 60 s, the right eye was to be photographed first and then the left, both eyes were to be photographed consecutively during the acquisition time, and the patient-role subject was to be photographed in a semi-dark room at an illuminance of 17.2 lux. Additionally, the subjects were allowed to dictate the patient-role subject’s fixation—eyelid opening, blinking, etc. In consideration of the load and risk to the patient-role subject, the first day of participation in the study for all subjects (Figure 1, “3 days before data acquisition”) was divided into several days. The subjects in the control group were given the same advice as in training groups 1 and 2 during viewing the videos taken during data acquisition. The smartphone used for data acquisition was an iPhone 7 with iOS version 16.7.7 (Apple Inc., Cupertino, CA, USA), 1080p resolution, and a frame rate of 30 fps.

**Figure 3:**
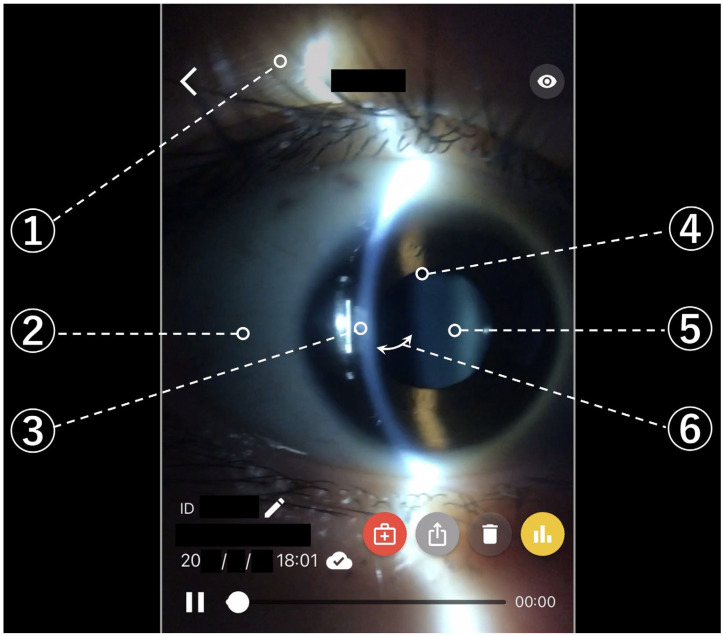
Screen of the filing system and judgment items (right eye). ① Eyelid, ② conjunctiva, ③ cornea, ④ pupil (including iris), ⑤ lens, and ⑥ anterior chamber depth (the curved arrow indicates the space between the posterior surface of the cornea and the anterior surface of the lens).

Videos taken by the subjects were uploaded to the SEC application filing system via cloud computing. The videos were judged by four ophthalmologists (M.N., T.M., K.T. and E.S.) through the SBSL video filing system and its telemedicine function, as explained previously. The video files were single-blinded so that the judges could not distinguish between the control group, training group 1, and training group 2, and the judges viewed the video files at a remote location different from where the videos were recorded. The items judged were the eyelid, conjunctiva, cornea, pupil (including iris), lens, and anterior chamber depth (Figure 3). The judges evaluated the videos recorded by the subjects based on the criterion that the video quality was sufficient to distinguish normal from abnormal findings for each tissue (i.e., each evaluated item) during clinical examination. For each item, the videos were assessed separately for the right and left eyes. The score, ranging from a minimum of 0 to a maximum of 4, was defined as the number of judges who determined that the videos of both the right and left eyes met this criterion.

### 2.4. Data Analysis

The interrater reliability of scores was determined by the intraclass correlation coefficient ICC (2, 1). The Kruskal–Wallis one-way analysis of variance (ANOVA) was used to analyze the differences among the scores, and post-hoc tests were conducted using the Mann–Whitney U test with Bonferroni correction for intergroup comparison when the ANOVA was significant. Training groups 1 and 2 were combined for the principal component analysis. R (https://www.r-project.org/ version 4.2.3 (accessed on 20 July 2023)) was used for statistical analysis. The significance level was set at 5%.

## 3. Results

The interrater reliability was rated as “poor” with an ICC = 0.407 (0.316–0.616), but was statistically significant (*p* < 0.001, ICC (2, 1)).

**Figure 4:**
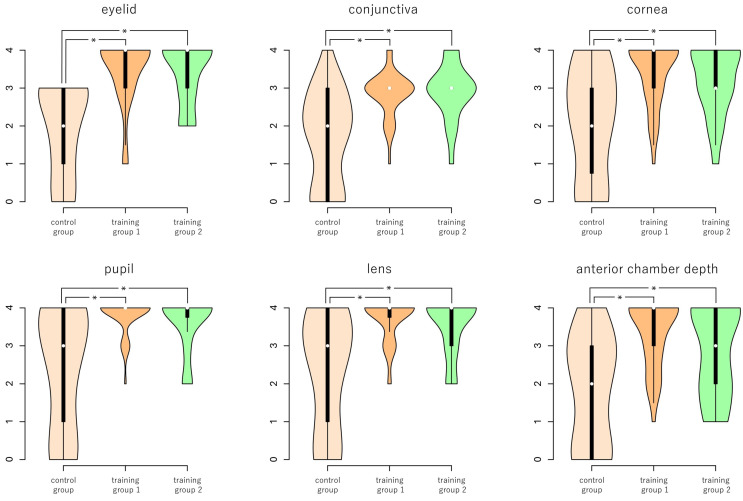
Comparison of scores between groups. The upper edge of the violin plot indicates the upper adjacent value, the lower edge indicates the lower adjacent value, the white circle within the black rectangle in the violin plot indicates the median, the upper edge indicates the third quartile, and the lower edge indicates the first quartile. * indicates *p* < 0.01.

ANOVA of the scores also showed significant differences in all the items (eyelid, conjunctiva, cornea, pupil, lens, and anterior chamber depth, *p* = 9.84 × 10^−7^, 1.50 × 10^−6^, 4.69 × 10^−5^, 3.58 × 10^−6^, 1.22 × 10^−6^, and 2.25 × 10^−5^, Kruskal–Wallis one-way ANOVA, respectively). As shown in Figure 4, comparisons between the three groups revealed significantly lower scores in the control group for all items between the control group and training group 1, and between the control group and training group 2, and not significant between training group 1 and training group 2 (for the control group and training group 1, control group and training group 2, and training group 1 and training group 2, *p* = 7.25 × 10^−6^, 1.25 × 10^−5^, and 1.00 for the eyelids, *p* = 2.64 × 10^−5^, 7.50 × 10^−6^, and 1.00 for the conjunctiva, *p* = 1.60 × 10^−4^, 4.28 × 10^−4^, and 1.00 for the cornea, *p* = 1.38 × 10^−5^, 6.50 × 10^−5^, and 1.00 for the pupil, *p* = 6.77 × 10^−6^, 2.01 × 10^−5^, and 1.00 for the lens, and *p* = 1.55 × 10^−5^, 0.006, and 0.22 for the anterior chamber depth, respectively, Mann–Whitney U test with Bonferroni correction).

Principal component analysis was performed on 80 eyes of 40 subjects in training group 1 and training group 2, whose scores on each criterion were not significantly different. The eigenvalue of the first PC was 4.3418, and the proportion of variance was 74.36%. On the other hand, the second PC had an eigenvalue of 0.6424 and a proportion of variance of 10.71%, and the subsequent third to sixth PCs also had eigenvalues below one and a contribution of less than 10% (Table 2).

## 4. Discussion

Ophthalmic examination instruments using smartphones have developed rapidly in recent years, enabling ophthalmic evaluations and meeting the demands of diagnostic necessities outside of ophthalmic clinical practice [24,25,26]. SBSL research is also emerging. For example, some studies involve SBSL design [2], interobserver reproducibility of tear break-up time [27], cataract diagnosis [19], epidemiological studies on remote islands [17], and consulting services for mainland Japan [18]. On the other hand, SBSLs are operated by general practitioners [17,18], nurses [19], non-ophthalmologists [27], and technicians [2], and the need for SBSL education has not been adequately addressed. In this study, we conducted a pilot study on coaching for non-ophthalmologists and non-medical personnel to obtain good quality anterior segment findings in a short period of time.

### 4.1. Validity of the Coaching Approach Used in This Study

Collis et al. [23] reported that after medical students watched a 22 min study video on the stationary slit-lamp microscope followed by 35 min of practice (10 min for the model eye and 25 min for the student partner), their skill scores were higher than those of medical students who received direct instruction without the video learning resources. Shikino et al. [28] also reported that medical students, who had no experience in clinical clerkship rotation in ophthalmology but who had experience in fundus examination, were given a 10 min video learning period for 48 h regarding a demonstration of fundus examination. The results were consistent with those of the present study. The difference in the learning process between these previous studies and the present study is that the present study provided direct advice to the training group that conducted the video study. Bursztyn et al. [29] had paraprofessionals (medical assistants, optometrists, or resident physicians) with no experience or training in fundus photography take a brief educational session and then had them take photographs of the optic disc using a handheld nonmydriatic fundus camera, and examined the sensitivity and specificity of the images for detecting normal optic discs and disc edema. Lamirel et al. [30] further examined the quality of fundus photographs taken by nurses in the emergency department based on materials from a stationary fundus camera with short training periods (written materials, 15 to 30 min of training using the camera, and feedback from researchers). Both of these previous studies indicate that non-ophthalmologists or medical students need to receive direct lectures through some kind of educational session or training to acquire ophthalmologic examination skills. Based on these reports, we considered it impractical not to provide some feedback to the subjects in training groups 1 and 2 in the coaching method used in this study, and we provided an opportunity for advice during the trial phase of the coaching process.

The coaching period in this study was 3 days. Succar et al. [31] reported that the Virtual Ophthalmology Clinic was incorporated into a clinical ophthalmology rotation of up to 10 days in medical school education, and that test scores improved significantly in the group that received the knowledge-based test before and after the rotation compared to the group that did not receive the Virtual Ophthalmology Clinic. The Virtual Ophthalmology Clinic was incorporated into a clinical ophthalmology rotation of up to 10 days in medical school education, and test scores were significantly improved in that group compared with the group that did not receive the Virtual Ophthalmology Clinic. However, we believe that the duration of coaching designed in this study is appropriate for various use scenarios, such as telemedicine and medical assistance, in countries with limited medical resources, and in SBSL use.

### 4.2. Effectiveness of SBSL-Based Coaching and Key Instructional Elements to Be Emphasized

In this study, a group of students with a one full year of orthoptic education (training group 1) and a group of students with just one month of medical education (training group 2) were set up to use the SBSL to evaluate the necessity of specialized ophthalmology education. In addition, in order to compare the effect of coaching, students with exactly the same orthoptic education as training group 1 received only written explanations and observed the operation of the SBSL (control group). The scores of both training groups 1 and 2 were better than those of the control group. Furthermore, there was no significant difference between the scores of training group 1 and 2, suggesting the proposed coaching method resulted in the equivalent effects on students regardless of the ophthalmologic expertise and ophthalmologic examination skills learned over the course of one year. Duong et al. [19] classified the degree of opacity of nuclear cataracts from slit images taken by an optometrist and a nurse using an SBSL by Buratto grading and WHO classification, and examined the interobserver reliability and accuracy of each classification. The authors reported that “Both (two other optometrists) were as well as received prior training to ensure consistency in grading.” However, in other previous studies in which an SBSL was used by non-ophthalmologists, even the existence of prior training was not specified [2,17,18,19,27]. Therefore, this study is the first report on the validation of the effectiveness of SBSL-based learning methods. As the main interpretation, we were able to suggest that the coaching method proposed in this study may have sufficient potential to build a certain foundation of imaging skills even for those with no to a poor ophthalmological background.

**Figure 5:**
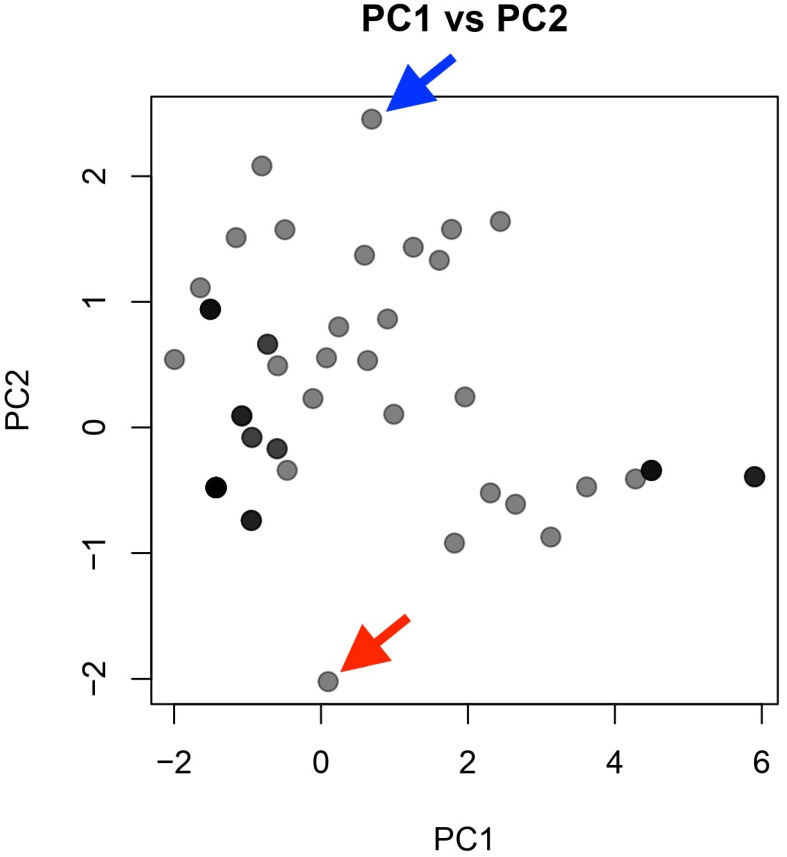
Biplot of PC1 and PC2. Every symbol represents an eye. Color indicates the number of symbols at the same location. Blue arrow: Typical subject good at focusing depth imaging but not good at conjunctiva imaging. Red arrow: Typical subject good at conjunctiva imaging but not good at anterior chamber depth imaging.

However, even among the coached subjects, not all the judges judged that all items could be used in medical practice. Therefore, a principal component analysis was performed, integrating the scores of training groups 1 and 2 (Table 2). The results showed that PC1 accounted for 74.36% of the contribution. The principal component loading was highest for the lenses (−0.4495), followed by the pupil (−0.4405), cornea (−0.4198), and eyelid (−0.3907), indicating that training groups 1 and 2 could take well-balanced images from the eyelid to lens. Despite the contribution rate being low (10.71%), PC2’s principal component loading was lowest in the conjunctiva (−0.5585) and highest in anterior chamber depth (0.7780), suggesting a possible tendency that subjects might be characterized by their favorite focusing depth; some subjects had good conjunctiva scores but poor anterior chamber depth (and vice versa) (Figure 5). However, because no direct behavioral data related to focusing strategy or device manipulation were collected, this interpretation should be regarded as speculative. There may be two plausible explanations. First, the subjects good at conjunctival imaging tended to focus on only the superficial parts of the eye and not notice the deeper parts, and vice versa. Second, if the subject initially focused on the corneal center and then rotated the slit light horizontally around the vertical axis of the SBSL to observe both sides of the conjunctiva, the conjunctiva might be out of focus due to the limited working distance of the SBSL. In this study, we did not measure the direction and/or position of the SBSL. Therefore, the possibility remains to be further investigated. In other words, to observe the anterior segmentation clearly enough for medical practice, it is necessary to take comprehensive images of every part of the anterior segment. It may be important to inform novice SBSL users of this point. In addition, we emphasized, among other specific advice for ensuring working distance, that when moving the SBSL horizontally, the SBSL should be moved parallel to the forehead without rotating it.

### 4.3. Future Directions and Limitations of This Study

The greatest advantage of SBSL learning is that the recorded data can be shared in real time and can be interactively exchanged even in remote locations [2,17,18]. In this study, explanations and advice were provided to the subjects in person, but SBSL coaching and advice can also be provided via online conferencing as long as an Internet environment such as Wi-Fi is available. In the future, we will examine the learning effects of coaching for those who have no ophthalmology background, taking into account the coaching issues which arose in the findings in this study.

The safety of the light source [32] and the device itself [9], which are concerns in the use of SBSLs, has already been confirmed. The SBSL used in this study has demonstrated comparable accuracy, reproducibility [33], and interobserver reliability [27,33] when compared to stationary devices. Furthermore, the SBSL has been used in the treatment of common diseases, such as allergic conjunctival diseases [14], for which multiple departments may be involved. Additionally, this study may provide useful knowledge that will serve as a basis for considering the application of SBSLs beyond the field of ophthalmology.

There are several limitations to this case. Firstly, the sample size is small. Additionally, the subjects were orthoptic students, and their characteristics could be biased. Therefore, in the future, we will increase the number of cases and, referring to studies on skill upskilling for smartphone-based fundus imaging among ophthalmic assistants and ophthalmologists [34], examine the results of medical professionals, medical students, and clinicians involved in departments other than ophthalmology as well. Secondly, in this study we tried to examine the effect of basic medical courses (e.g., anatomy and physiology) on the acquisition of SBSL imaging by comparing training groups 1 and 2, rather than a randomized control test (RCT) for it. Therefore, our results on the acquisition of SBSL imaging skill might be underestimated. We also did not employ double-blind testing but did single-blind tests. However, the artefact caused by this limitation may be minimal, because the researchers did not observe the subjects during image acquisition. To address these issues, we will plan RCTs with diverse users. Thirdly, the study was conducted using only the slit-light illumination method. Since we aimed for pure skill evaluation in this study, we selected the same patient-role subject. We judged that it would be burdensome for the subjects to be photographed under all conditions of fluorescein staining using white diffuse light and blue light. Therefore, we imposed only using the SBSL using slit-light inspections. Furthermore, we did not conduct a pre-skill assessment on the slit-lamp microscope, so it remains unclear whether coaching improved skills; additionally, we did not conduct interviews regarding the coaching or the use of the SBSL, nor did we evaluate the long-term retention of the skills acquired by the subjects. We plan to continue our study based on a methodology that incorporates these aspects.

## 5. Conclusions

We investigated the effect of coaching for the smartphone-based slit-lamp microscope (SEC). The inexperienced participants had better usage abilities due to the tutorial video, the 30 min trial, and feedback from experienced SBSL users, in comparison with participants with over a year of regular ophthalmological medical exposure. These results suggested that our coaching methods for the SBSL may be effective for people without an ophthalmological background. On the other hand, when coaching using an SBSL, it should be emphasized that the anterior segmentation must be comprehensively imaged while maintaining an appropriate working distance.

## Figures and Tables

**Table 1:** Content about the tutorial video for the SEC.

Term	Description
1. How to install SEC attachments	Correct orientation of attachments to smartphone.
2. Attachments and 3 types of light	Introduction to slit light and suitable cases for observation. Introduction to white diffuse light and suitable cases for observation. Introduction to blue diffuse light and suitable cases for observation.
3. How to use the application	Installing the application required for operation. How to log in to the application.
4. Shooting flow and points to keep in mind	Shooting procedures and operation. How to hold the SEC. - When observing the patient’s right eye, hold the SEC with the right hand and place the left hand on the patient’s forehead and the SEC to create a shadow (when observing the left eye, these left and right hands have opposite roles). - The working distance from the lens of the attachment to the corneal apex should be 4.0 cm. - Hold the SEC parallel to the patient’s forehead. - The angle of view during imaging should be such that the cornea occupies 80% of the image. - The slit light should be centered on the cornea. Directing the patient to open the eyelids and look at the patient.
5. Other	How to preview the video you shot.

**Table 2:** Principal component analysis of scores integrating training group 1 and training group 2.

	**Principal Component**					
	**PC 1**	**PC 2**	**PC 3**	**PC 4**	**PC 5**	**PC 6**
Eigenvalue	4.3418	0.6424	0.4025	0.3068	0.2086	0.0980
Proportion of variance	74.36%	10.71%	6.71%	5.11%	3.48%	1.63%
Cumulative proportion	74.36%	83.07%	89.78%	94.89%	98.37%	100.00%
	**Principal Component Loading**					
	PC 1	PC 2	PC 3	PC 4	PC 5	PC 6
Eyelid	−0.3907	−0.0756	0.8990	−0.1240	−0.1327	0.0209
Conjunctiva	−0.3899	−0.5585	−0.0685	0.5283	0.4957	−0.0804
Cornea	−0.4198	0.1613	−0.1813	−0.6813	0.5476	−0.0310
Pupil	−0.4405	−0.2255	−0.3031	−0.0862	−0.4403	0.6797
Lens	−0.4495	0.0133	−0.2496	−0.0073	−0.4804	−0.7103
Anterior chamber depth	−0.3508	0.7780	−0.0077	0.4836	0.1100	0.1599

## Data Availability

The original contributions presented in this study are included in the article. Further inquiries can be directed to the corresponding author.

## Abbreviations

The following abbreviations are used in this manuscript:

SBSL	Smartphone-based slit-lamp microscope
SEC	Smart Eye Camera
